# Transcriptomic Analyses Reveal the Protective Immune Regulation of Conjugated Linoleic Acids in Sheep Ruminal Epithelial Cells

**DOI:** 10.3389/fphys.2020.588082

**Published:** 2020-10-29

**Authors:** Chunlei Yang, Wei Lan, Shijie Ye, Binna Zhu, Zhengwei Fu

**Affiliations:** ^1^College of Biotechnology and Bioengineering, Zhejiang University of Technology, Hangzhou, China; ^2^College of Animal Sciences, Zhejiang University, Hangzhou, China

**Keywords:** ruminal epithelial cells, immortalization, CLA, immune regulation, RNA sequencing

## Abstract

The ruminal epithelium is continuously challenged by antigens released by the lysis of dead microbial cells within the rumen. However, the innate immune system of the ruminal epithelium can almost always actively respond to these challenges. The cross talk between the ruminal microbiota and innate immune cells in the ruminal epithelium has been suggested to play an important role in sustaining the balance of immune tolerance and inflammatory response in the rumen. We hypothesized that conjugated linoleic acid (CLA), a functional microbial metabolite in the rumen, may contribute to the immune regulation in rumen epithelial cells (RECs); therefore, we first established an immortal REC line and then investigated the regulatory effects of CLA on the immune responses in these RECs. The results showed that long-term REC cultures were successfully established via SV40T-induced immortalization. Transcriptome analysis showed that a 100 μM CLA mixture consisting of 50:50 *cis*-9, *trans*-11:*trans*-10, *cis*-12 CLA significantly downregulated the expression of the inflammatory response-related genes TNF-α, IL-6, CX3CL1, IRF1, ICAM1 and EDN1, and upregulated the expression of the cell proliferation-related genes FGF7, FGF21, EREG, AREG and HBEGF and the lipid metabolism-related genes PLIN2, CPT1A, ANGPTL4, ABHD5 and SREBF1 in the RECs upon LPS stimulation. Correspondingly, the GO terms regulation of cell adhesion, response to stimulus and cytokine production and KEGG pathways TNF and HIF-1 signaling, ECM-receptor interaction and cell adhesion molecules were identified for the significantly downregulated genes, while the GO terms epithelial cell proliferation and regulation of epithelial cell migration and the KEGG pathways PPAR, ErbB and adipocytokine signaling were identified for the RECs with significantly upregulated CLA-pretreated genes upon LPS stimulation. These findings revealed that CLA conferred protective immunity onto the RECs by inhibiting proinflammatory processes, promoting cell proliferation and regulating lipid metabolism related to the immune response.

## Introduction

For ruminants, the ruminal epithelium plays an important role not only in nutrient uptake but also as an important barrier protecting host animals against microbial invasion and mechanical damage (Aschenbach et al., 2019). The free lipopolysaccharide (LPS) derived from shedding gram-negative bacteria or the lysis of dead microbial cells within the rumen leads to great challenges for the ruminal epithelium, especially when coinciding with metabolic disorders, such as ruminal acidosis (Mao et al., 2016). Although the innate immune system of the ruminal epithelium can almost always respond to challenges actively, the molecular mechanisms have not been fully clarified. Rumen function is inhibited by diverse microbiota, and the cross talk of microbiota and innate immune cells in the ruminal epithelium has been suggested to substantially contribute to the balance of immune tolerance and inflammatory response in the rumen, which is partly dependent on metabolites (Minuti et al., 2015; Shen et al., 2019).

Conjugated linoleic acid (CLA) is a mixture of positional and geometrical isomers of linoleic acid with conjugated double bonds that can be naturally produced during the microbial biohydrogenation process in the rumen. Among the broad range of CLA isomers, *cis*-9, *trans*-11 CLA and *trans*-10, *cis*-12-CLA have been the most studied bioactive isomers (Bruen et al., 2017), and many healthy characteristics have been associated with the results of interactions between these two major isomers, such as anti-obesogenic, anti-carcinogenic, anti-inflammatory and immune enhancement properties (Kim et al., 2016; Viladomiu et al., 2016; Garibay-Nieto et al., 2017). Previous studies on ruminants found that the addition of *cis*-9, *trans*-11 CLA and *trans*-10, *cis*-12 CLA can modify the fatty acid profile of sheep’s milk and beef (Schiavon et al., 2011; Pellattiero et al., 2015), affect the fatty acid metabolism pathways in ovine ruminal epithelial cells (Masur et al., 2016) and lower the milk fat content and improve the energy balance in dairy cows during early lactation (Qin et al., 2018). Basiricò et al. (2017) also found that supplementation of *cis*-9, *trans*-11 CLA and *trans*-10, *cis*-12 CLA protected bovine mammary epithelial cells against H_2_O_2_-induced oxidative damage. However, information about the role of CLA in regulating the immune responses of the ruminal epithelium and related molecular mechanisms is limited.

Several mechanisms have been indicated for the positive effects of CLA on the immune response. The most well-known pathway is the peroxisome proliferator-activated receptor (PPAR)-dependent pathway because of the high similarity of CLA and PPAR ligands (Yuan et al., 2015). PPARs can transduce a variety of inflammatory and nutritional signals into an ordered set of cellular responses at the gene transcriptional level (Daynes and Jones, 2002). In addition, the ability of CLA to regulate lipid metabolism, cell survival, and signal activation, which are involved in immune processes, has also been suggested to be closely related to the CLA-induced immunoregulatory activity (Chen et al., 2019; Moreira et al., 2019; Mohammadi et al., 2020). RNA plays an established essential role in multiple biological processes, and RNA sequencing has allowed in-depth study into gene changes and alternative splicing in many cell populations and has enabled the possibility of detecting novel transcripts (Rai et al., 2018). To elucidate the possible immunoregulatory effects of CLA on rumen epithelial cells and related molecular mechanisms, we established long-term cultures of ruminal epithelial cells (RECs) via SV40T-induced immortalization. Then, the changed gene expression and function of the RECs, which were treated with 50:50 mixtures of *cis*-9, *trans*-11: *trans*-10, *cis*-12 CLA under inflammatory conditions, were investigated using the RNA-sequencing method.

## Materials and Methods

### Isolation and Culture of Primary Ruminal Epithelial Cells From Sheep

The experiments using lambs in this study were performed in compliance with the guidelines of the Institutional Animal Care and Use Committee of Zhejiang University of Technology. Rumen epithelial tissue was obtained from two young Hu lambs that were three days old and fed ewe’s milk. The isolation and culture of the primary RECs were performed as described previously with minor modifications (Klotz et al., 2001). The collected tissues were repeatedly washed with ice-cold PBS containing 200 U/ml penicillin and 200 μg/ml streptomycin until the solution was clear. Then, the tissues were minced into approximately 1 cm^2^ pieces and continuously washed with PBS containing 2% penicillin/streptomycin, 1% gentamicin and 1% amphotericin B until the supernatant was clear. The minced epithelium pieces were placed into a digestion flask containing 0.25% trypsin-0.02% EDTA in Hanks’ balanced salt solution. The digestion was performed in warm water bath with slow shaking for 10 min at 37°C. The digestion solution was discarded and replaced with fresh solution two or three times to remove the stratum corneum epithelium. The remaining epithelial tissues were then digested using the same method, with the process performed from four to six times with the digestion solution collected each time. Termination of the trypsinization with FBS was performed after cell harvest. The harvested cell solution was filtered through a 0.15-mm nylon mesh and then centrifuged at 300 × *g* for 5 min at 4°C, and then, the supernatant was discarded. Cell pellets were resuspended in DMEM containing 2% FBS, 1% penicillin/streptomycin and 1% epithelial cell additive, including 25 ng/ml epidermal growth factor, 100 ng/ml hydrocortisone, 10 μg/ml human insulin, 5 μg/ml transferrin, 87 ng/ml cholera toxin, and 1.3 × 10^–2^ ng/ml triiodothyronine, and cultured at 37°C with 5% CO_2_ for 1 h. After incubation, the unattached cell supernatant was discarded and replaced with new medium, and the cells were grown to confluency, which required as much as 48 h.

### Immortalization of Lamb Rumen Epithelial Cells

Primary RECs were transfected with lentiviruses that express SV40 large T antigen and a puromycin resistance gene marker (Supplementary Table 1). After 12 h, the viral supernatant was removed, and the cells were continuously cultured in new medium until they were confluent. Then, the cells were aliquoted at a concentration of 5 × 10^4^ into each well of 24-well plate and cultured overnight. Subsequently, the supernatant was removed and replaced with new medium containing 2 μg/ml puromycin. The cells were continuously cultured, and the cell survival ratio was determined every day. The cells that survived through 3 days of incubation with puromycin were considered successfully transfected cells. These successfully transfected cells were subcultured for at least 30 passages to obtain immortal RECs.

### Identification of Immortalized Ovine Rumen Epithelial Cells

To validate the origin of the immortal RECs, immunocytochemical staining was performed to identify the presence of cytokeratin. The cells were fixed with 4% PFA for 0.5 h at 4°C, washed three times with PBS and incubated overnight at 4°C. Subsequently, the cells were permeabilized for 2 h with 0.25% PBS-Triton X-100 that contained 10% serum. The cells were then incubated overnight with primary rabbit anti-cytokeratin 19 antibody (diluted 1:100) at 4°C and then incubated for 2 h with secondary Alexa Fluor 594 conjugated goat anti-rabbit IgG antibody (diluted 1:500) at room temperature in the dark, followed by 3 rinses with PBS for 5 min each time and dyed with DAPI (DAPI:PBS = 1:1000) for 5 min. After being rinsing again with PBS, the cells were visualized by confocal laser microscopy after treatment with Fluoromount-G.

### Cell Culture and Treatment

After culturing the immortalized RECs for 3 passages, the cells were seeded in 6-well cell culture plates and cultured until 70–80% confluent. Different concentrations of LPS (0.1, 1 and 10 μg/ml) were used to treat RECs for 3 h (Yoshioka et al., 2016) to select the minimum concentration to construct inflammation model in RECs. Subsequently, the cells were untreated (control group, CON) or treated with 0.1 μg/ml LPS for 3 h after they were pretreated with 100 μM of 50:50 mixtures of *cis*-9, *trans*-11:*trans*-10, *cis*-12 CLA (Sigma-Aldrich, Shanghai, China) for 24 h (CLA + LPS group, CLA + LPS) or not (LPS group, LPS) (Changhua et al., 2005; Saba et al., 2019). Each group consisted of 4 biological replicates, and each biological replicate had 3 technical replicates.

### Quantitative Real-Time PCR (qPCR)

Total RNA was extracted from all the treated RECs using the RNA PURE KIT (Aidlab Biotechnologies Co., Ltd., Beijing, China) according to the manufacturer’s protocol. Then cDNA synthesis was performed using the PrimeScript RT Reagent Kit (Takara). Subsequently, qPCR was performed with SYBR green in an ABI 7500 (Life Technologies, Singapore) using the following procedures: predenaturation at 95°C for 30 s, followed by 40 cycles of 95°C and 60°C for 5 and 34 s, respectively. The results were normalized to the expression of YWHAZ and GADPH using the 2^–ΔΔ*Ct*^ method. All the primers (Supplementary Table 2) were designed using the Basic Local Alignment Search Tool [BLAST; National Center for Biotechnology Information (NCBI), Bethesda, MD, United States].

### Illumina HiSeq mRNA Sequencing

After RNA extraction, the concentration and purity of the extracted RNA were determined using the Qubit^®^ RNA assay kit with a Qubit^®^2.0 fluorometer (Life Technologies, Carlsbad, CA, United States) and NanoPhotometer^®^ spectrophotometer (IMPLEN, Westlake Village, CA, United States), respectively. RNA integrity was measured using the RNA Nano 6000 assay kit with the Bioanalyzer 2100 system (Agilent Technologies, Santa Clara, CA, United States). A total of 3 μg of RNA per sample was used for library preparation using the NEBNext^®^ Ultra^TM^ RNA library prep kit for Illumina^®^ (NEB, United States) according to the manufacturer’s instructions. Paired-end sequencing (150 bp) was carried out using the Illumina HiSeq 2000 instrument, with a minimum depth of 40 million reads per sample obtained. The sequencing work was supported by the Beijing Novogene Biological Information Technology Co., LTD.

### Bioinformatics Analysis

Clean data were obtained from the raw data by removing the reads containing adapter and poly-*N* sequences and then mapped to the reference genome and gene model annotation files of *Ovis aries*^1^ using Hisat2v.2.0.5. The gene expression level (FPKM, fragments per kilobase of transcript per million fragments mapped) was calculated according to the length of the gene and read count mapped to this gene. The DESeq2 R package (1.16.1) was used to analyze the differential gene expression among the CON, LPS and CLA + LPS groups. The adjusted *P* value (*P*_adj_) was calculated using the Benjamini and Hochberg approach to control the false discovery rate. The thresholds of *P*_adj_ < 0.01 and fold change (FC) >1.5 were used to filter out differentially expressed genes (DEGs) (Han et al., 2019). Gene Ontology (GO) enrichment and Kyoto Encyclopedia of Genes and Genomes (KEGG) pathway analysis of the DEGs were performed with the GOseq R package and KOBAS software, respectively, to better understand their functional roles. GO or KEGG terms with *P* < 0.05 were considered significantly enriched with DEGs. Interaction networks of the DEGs were analyzed based on the STRING v.10.5 database^2^ to better understand the relationships between the proteins and genes identified. Hub genes (degree > 5) in the networks were determined by Cytoscape. The sequences obtained in this study were deposited in the NCBI Sequence Read Archive under accession number PRJNA648485.

### Data Analysis

The data for qPCR results are were reported as the means ± SEM. Comparisons between two groups were analyzed using unpaired Student’s *t*-test. *P* values <0.05 were considered statistically significant.

## Results

### Establishment and Characterization of the RECs

**FIGURE 1:**
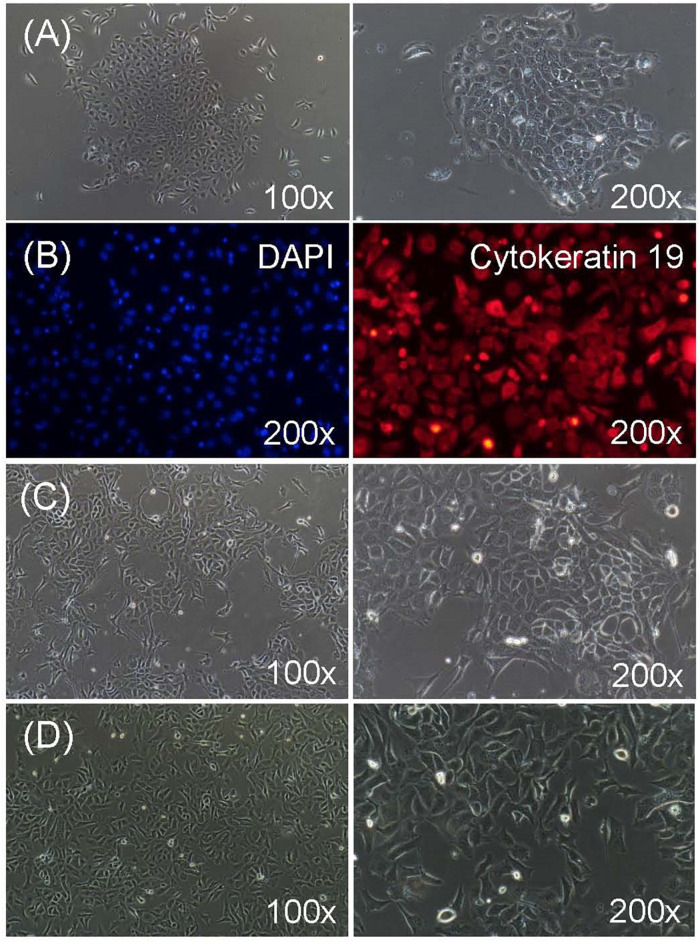
Characterization of ruminal epithelial cells from sheep (RECs). **(A)** Morphology of isolated primary RECs. The cell aggregates were well-attached in 24 h of culture. **(B)** Immunofluorescence staining of primary RECs. Cytokeratin 19: fluorescent image showing stained cytokeratin 19; DAPI: negative control when only secondary antibody was used; nuclei were stained with DAPI. **(C)** Morphology of successfully immortalized REC induced by SV40T after puromycin selection. **(D)** Morphology of immortal RECs after 30 passages of culture without showing any signs of senescence.

After digestion with 0.25% trypsin for 4 to 6 times, most of the RECs were successfully isolated from rumen tissues with the corneum removed. The isolated RECs adhered to the plastic substratum of the culture plate after 12 h of culture, and began to proliferate and generated clusters after 24 h of culture (Figure 1A). Typical characteristics of epithelial-like “cobblestone” morphology was observed for these newly isolated RECs. They also exhibited a strong immunopositive staining for cytoskeleton 19, which is a marker of epithelial cells, and no specific staining in the cells was observed for the negative control, which was exposed only to the secondary antibody (Figure 1B). To prevent the senescence of the primary RECs, immortal RECs were then established using SV40T (Figure 1C). The immortal RECs maintained the typical epithelial-like “cobblestone” morphology and could be cultured at least 30 passages without showing any signs of senescence (Figure 1D).

### Verification of the Inflammatory Models in RECs

**FIGURE 2:**
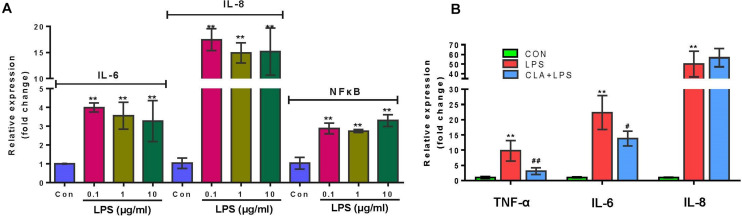
Verification of the pro-inflammatory effect of LPS and anti-inflammatory effect of CLA. **(A)** Relative gene expression of IL-6, IL-8 and NF-κB in RECs after stimulating with 0.1, 1, and 10 μg/ml LPS for 3 h respectively. **(B)** Relative gene expression of TNF-α, IL-6 and IL-8 in RECs with the 100 μM CLA pretreatment for 24 h during 0.1 μg/ml LPS stimulation. *indicates significant difference compared to the CON group (*P < 0.05, **P < 0.01), ^#^ indicates significant difference compared to the LPS group (^#^*P* < 0.05, ^##^*P* < 0.01).

The qPCR results indicated that the expression of IL-6, IL-8 and NF-κB in RECs was significantly increased with the treatment of LPS for 3 h at the concentration of 0.1, 1, and 10 μg/ml respectively (*P* < 0.01) (Figure 2A). Therefore, 0.1 μg/ml LPS was enough and further used to induce inflammation in RECs. The 100 μM CLA pretreatment for 24 h significantly suppressed the expression of TNF-α (*P* < 0.01) and IL-6 (*P* < 0.05) in RECs upon LPS (0.1 μg/ml) stimulation (Figure 2B).

### Overall Gene Expression Among Different Treatments

**FIGURE 3:**
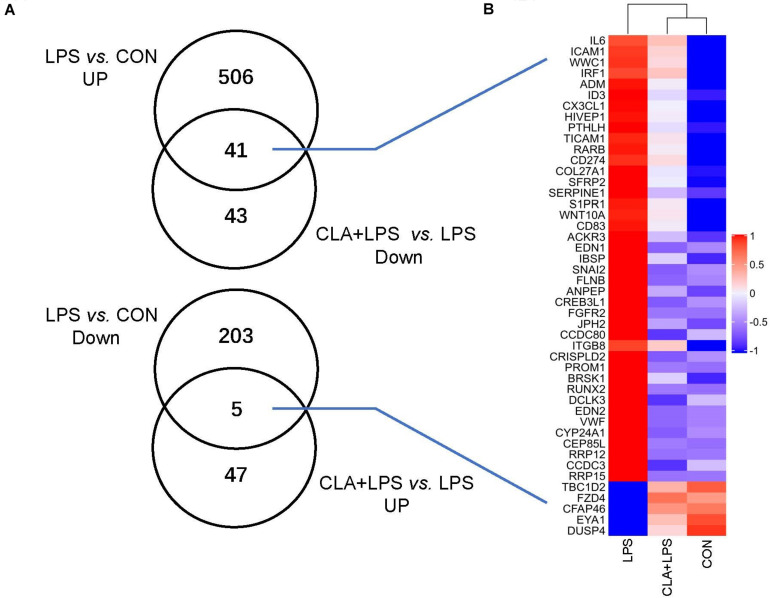
Overview of the differentially expressed genes (DEGs) after different treatments. **(A)** Venn diagrams of known DEGs based on comparisons of different groups. Cut-off for the differential expression criteria were *P*_adj_ < 0.01 and FC > 1.5. **(B)** Hierarchical clustering of the overlapping DEGs among different groups. The color code indicates highly expressed genes (red) and genes expressed at low levels (blue).

An average of 52.1 million clean reads per sample was obtained after raw data cleaning. Over 85.11% of the clean reads for each sample were mapped to the reference genome of *Ovis aries*. After calculating the FPKM of all the mapped genes in each sample, all results showed that treatments led to a similar gene expression distribution. After novel genes were removed, 547 genes were significantly upregulated and 208 were downregulated in the LPS group compared the expression levels in the CON group (FC > 1.5, *P*_adj_ < 0.01). Fifty-two genes were significantly upregulated, and 84 genes were downregulated in the CLA + LPS group compared with the LPS group. Specifically, 41 overlapping genes were significantly upregulated with LPS treatment alone (LPS group) and downregulated with CLA pretreatment during LPS stimulation (CLA + LPS group), while 5 overlapping genes were significantly downregulated with LPS treatment alone (LPS group) and upregulated with CLA pretreatment and LPS stimulation (CLA + LPS group) (Figure 3A). The hierarchical cluster analysis of these overlapping DEGs revealed a clear separation of the CON and CLA + LPS groups from the LPS group (Figure 3B).

### GO and KEGG Enrichment Analyses

**FIGURE 4:**
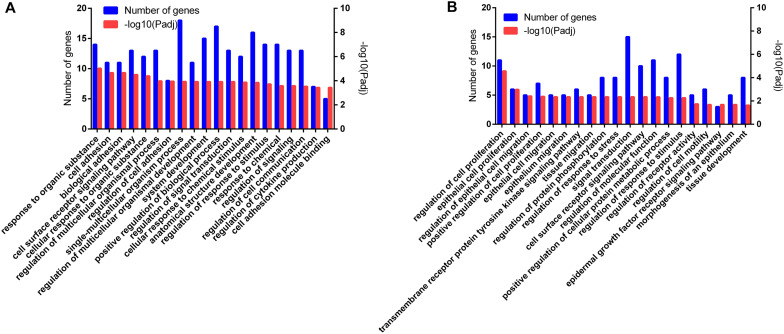
Functional annotation of the DEG genes according to the GO analyses. **(A)** GO enrichment analysis of the overlapping DEGs that were downregulated in the CLA + LPS group and upregulated in the LPS group. **(B)** GO enrichment analysis of the DEGs that were upregulated in the CLA + LPS group compared with gene expression in the LPS group. Only the top 20 GO terms are shown (*P*_adj_ < 0.01).

**FIGURE 5:**
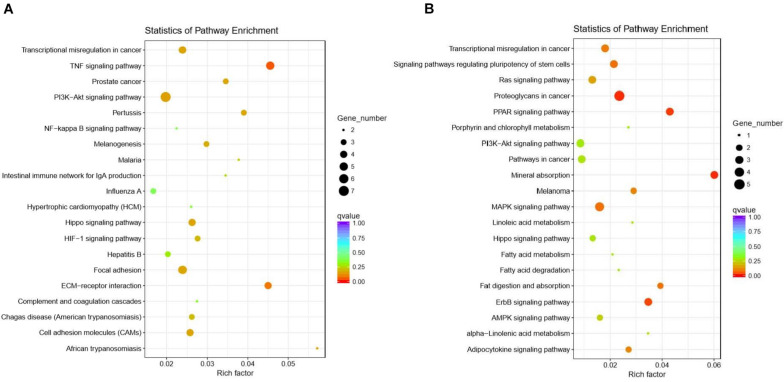
Functional annotation of the DEG genes according to the KEGG analyses. **(A)** KEGG enrichment analysis of the overlapping DEGs that were downregulated in the CLA + LPS group and upregulated in the LPS group. **(B)** KEGG enrichment analysis of the DEGs that were upregulated in the CLA + LPS group compared with the gene expression in the LPS group. Circles represent the numbers of enriched genes, and colors indicate the *P*_adj_ values.

In total, 932 GO terms were significantly (*P* < 0.05) enriched with the overlapping genes that were downregulated in the CLA + LPS group and upregulated in the LPS group. GO analysis revealed that the overlapping genes were primarily enriched in the biological process of cell adhesion, cell surface receptor signaling pathway, regulation of signal transduction, cellular response to chemical stimulus, regulation of response to stimulus, regulation of cytokine production (Figure 4A). KEGG analysis showed that 15 pathways were significantly (*P* < 0.05) enriched with these genes, including the TNF and HIF-1 signaling pathways, ECM-receptor interaction, cell adhesion molecules (CAMs) (Figure 5A).

For the genes that were upregulated in the CLA + LPS group compared with the LPS group, 637 GO terms were significantly (*P* < 0.05) enriched, primarily in the following biological process categories: regulation of cell proliferation, epithelial cell proliferation, regulation of epithelial cell migration, regulation of response to stress and epidermal growth factor receptor signaling pathway (Figure 4B). According to the KEGG analysis, the upregulated genes were significantly enriched to 11 pathways (*P* < 0.05), which primarily involved in PPAR, ErbB and MAPK signaling pathways, signaling pathways regulating pluripotency of stem cells, and adipocytokine signaling pathway (Figure 5B).

### Protein-Protein Interaction Analysis

**FIGURE 6:**
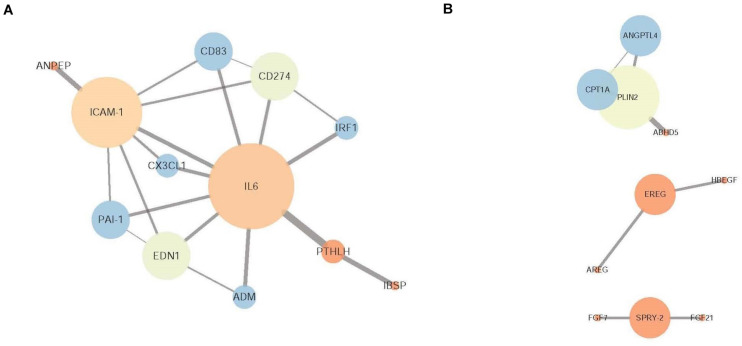
Protein-protein interaction (PPI) network of DEGs. **(A)** PPI network of the overlapping DEGs that were downregulated in the CLA + LPS group and upregulated in the LPS group. **(B)** PPI network of the DEGs that were upregulated in the CLA + LPS group compared with the expression level in the LPS group. The node color is determined by the clustering coefficient (low values are red, and high values are blue), and the node size is proportional to the number of degrees. Each edge represents the interaction (a thicker edge indicates lower betweenness).

In total, 42 nodes and 39 edges were established in the protein-protein interaction (PPI) network for the overlapping genes that were significantly downregulated with the pretreatment of CLA and upregulated by LPS stimulation (confidence score >0.4) (Supplementary Figure 1). The proinflammatory cytokine IL-6 and intercellular adhesion molecule ICAM1 were determined to be hub genes after the degree centrality analysis of the PPI network was performed (degree >5) (Figure 6A). Of the genes that were upregulated with CLA pretreatment and LPS stimulation compared with their expression in the LPS group, a total of 52 nodes and 12 edges were established in the PPI network (confidence score > 0.4) (Supplementary Figure 2), and the genes PLIN2 (degree = 3), CPT1A (degree = 2), SPRY2 (degree = 2), EREG (degree = 2) and ANGPTL4 (degree = 2) occupied core regulatory positions in the networks after the degree centrality analysis was performed (Figure 6B).

### Changes in the Expression of Immune Response-, Cell Growth-, and Fatty Acid Metabolism-Related Genes

**FIGURE 7:**
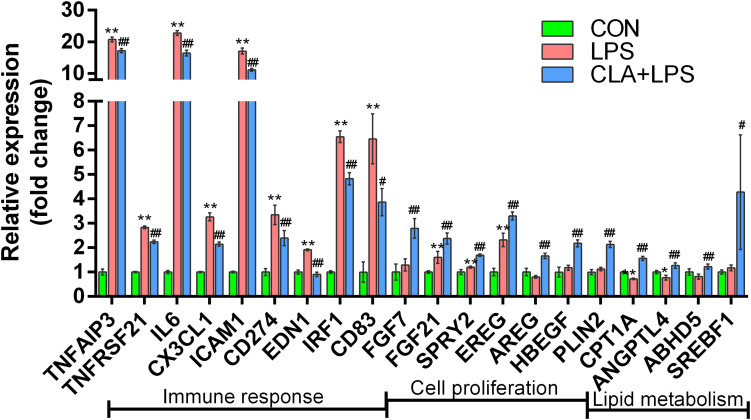
Gene expression analysis of the DEGs involved in the immune response, cell proliferation and lipid metabolism among different treatments. Gene names are shown on the *x*-axis, and the fold change value is shown on the *y*-axis. * indicates significant difference compared to the CON group (*P < 0.05, **P < 0.01), ^#^indicates significant difference compared to the LPS group (^#^*P* < 0.05, ^##^*P* < 0.01).

According to the gene expression level, the inflammatory response-related genes TNFAIP3, TNFRSF21, IL-6, CX3CL1, ICAM1, CD274, EDN1, IRF1 and CD83 were significantly upregulated in the LPS group compared with those in the CON group; however, pretreatment with CLA significantly downregulated the expression of these genes during LPS stimulation (CLA + LPS vs. LPS) (*P* < 0.05). In addition, pretreatment with CLA significantly upregulated the cell proliferation-related genes FGF7, FGF21, SPRY2, EREG, AREG and HBEGF and the lipid metabolism-related genes PLIN2, CPT1A, ANGPTL4, ABHD5 and SREBF1 (*P* < 0.05) (Figure 7).

## Discussion

In the present study, immortalized sheep RECs were successfully established and verified for the utilization in an investigation into the regulatory effects of the ruminal microbial product CLA on the immune response of ruminal epithelium upon challenges. The immortalized sheep RECs established in our study were cultured through at least 30 passages without presenting any signs of senescence, which prevented large numbers of programmed cell deaths during the long-term *in vitro* culture of primary epithelial cell research models (Zhan et al., 2017). An immunofluorescence analysis of cytokeratin 19 was performed to confirm that the immortalized sheep RECs were epithelial in origin. Cytokeratin 19 is usually utilized to differentiate cells of epithelial origin cells from stellate cells, and the presence of cytokeratin 19 in gastrointestinal epithelium has also been found in studies by Stammberger and Baczako (1999).

Due to the continuous production of CLA during the ruminal microbial biohydrogenation process, ruminal epithelial cells are directly exposed to a certain amount of CLA (Masur et al., 2016), and the positive immunoregulatory effect of CLA on gastrointestinal barrier health has been extensively verified in both animal and human studies (Chen et al., 2019; Coelho et al., 2019). Considering that the rumen immune system must continuously face challenges from antigens of lysed dead microbial cells or LPS from the shedding gram-negative bacteria within the rumen, we hypothesized that CLA may contribute to the immune response of the ruminal epithelium. In this study, we demonstrated that 100 μM *cis*-9, *trans*-11-CLA:*trans*-10, *cis*-12-CLA CLA (1:1) exerted inhibitory effects on the production of the proinflammatory cytokines TNF-α and IL-6, TNF receptor TNFAIP3 and TNFRSF21, chemokine CX3CL1, cell adhesion molecule ICAM1, interferon regulatory factor IRF1, endothelin-1 (EDN1), immunoglobulin superfamily member (CD83) and programmed cell death-ligand 1 (CD274) by ruminal epithelial cells induced by LPS stimulation. It is well known that inflammation is characterized by the recruitment of leukocytes and the largely choreographed release of cytokines (Ernandez and Mayadas, 2009). For example, TNF can induce the expression of endothelial adhesion molecules that support leukocyte-endothelial interactions and stimulate chemokine production that promotes the activation and transmigration of leukocytes (Ward-Kavanagh et al., 2016). Similarly, the regulation of IL-6 on neutrophil-activating chemokines, adhesion molecules and apoptotic regulators was also extensively discussed (Jones and Jenkins, 2018). Furthermore, the TNF receptor was reported to induce autocrine IRF1-dependent interferon-β signaling to promote monocyte recruitment during the TNF-induced inflammatory response of endothelial cells (Venkatesh et al., 2013). The positive role of IRF1 as an important transcriptional regulator in the inflammatory process has also been verified in other studies (Langlais et al., 2016). The proinflammatory responses of TNF have also been attributed to its effect on the vascular endothelium (Bradley, 2008). EDN1 is a vasoconstrictor that has been found to be closely related to IBD development (Murch et al., 1992), and it can also lead to intestinal oxidant stress and mucosal dysfunction by inducing polymorphonuclear leukocyte infiltration and adhesion molecule expression (Oktar et al., 2000). The positive correlation of TNF and EDN1 was found in the studies of Denisenko et al. (2019). Therefore, our study showed the positive immunoregulatory effect of CLA against LPS-induced inflammation in the rumen as indicated by the inhibition of proinflammatory cytokines, adhesion molecules, and the chemokine-induced cascade amplification response.

We used the RNA-Seq transcriptome to investigate the anti-inflammatory mechanism of CLA and found that pretreatment with CLA downregulated many signaling pathways, including the TNF signaling pathway, cell adhesion molecules (CAMs), extracellular matrix (ECM)-receptor interaction and the HIF-1 signaling pathway. TNF signaling is able to induce a wide range of intracellular inflammatory signaling pathways and promote the production of proinflammatory cytokines and chemokines (Ward-Kavanagh et al., 2016). Upregulated TNF expression has also been found in intestinal inflammation research models (Yan et al., 2019). CAMs express surface glycoproteins that can be used to classify the cells into families of integrins, cadherins, selectins and immunoglobulin CAMs. They have been found to be involved in mediating cell-cell interactions, binding leukocytes and other cells to the ECM, and in cell-cell and transmembrane signaling, and they play important roles in inflammation and the immune response (Ayuk et al., 2016). The interaction of leukocytes with ECM components controls various inflammatory processes, including leukocyte retention, accumulation, proliferation, migration, differentiation and activation (Wight et al., 2017). Improved ECM-receptor interactions have also been found in models of intestinal inflammation (Avula et al., 2012). In addition, HIF is an important transcriptional factor that acts downstream of multiple metabolic and immune signals and can in turn regulate host metabolism and immunity (Halligan et al., 2016). The cross talk between HIF and NF−κB in the regulation of the immune response among different immune cell types, such as B and T cells, macrophages, and neutrophils, has been extensively discussed (Dignazio et al., 2016), and the important role of HIF in stimulating the expression of LPS-induced inflammatory cytokines has also been proven (Peyssonnaux et al., 2007). Our results showed that the ErbB and MAPK signaling pathway, which are closely related to cell proliferation and migration (Kataria et al., 2019), were significantly upregulated by pretreatment with CLA. In accordance with these changes, the cell proliferation-related genes FGF7 (Pinto et al., 2011), FGF21 (Guo et al., 2018), EREG (Liu et al., 2017), AREG (Ko et al., 2020), HBEGF (Kamanga-Sollo et al., 2014) and SPRY2 (Zhao et al., 2018) were also upregulated. Cell proliferation and migration are key elements in epithelial barrier injury repair (Iizuka and Konno, 2011). Corresponding to this phenomenon, the regulation of epithelial cell proliferation and migration, as well as signaling pathways regulating pluripotency of stem cells, epidermal growth factor receptor signaling pathway were all promoted by CLA treatment. In addition, CLA treatment significantly enhanced the PPAR signaling pathway and improved the expression of PLIN2 (a target gene of PPARγ) (Kim et al., 2019) in our study, a finding that is consistent with the findings that CLA has the ability to bind to and activate PPARs due to its functional similarities to the ligand of PPARγ (Yuan et al., 2015). PPARγ plays a beneficial role in intestinal inflammation regulation and has been widely used in IBD therapy due to its ability to inhibit the activities of many inflammatory mediators, such as NF-κB and activator protein 1 (AP1) (Daynes and Jones, 2002). Therefore, the inhibition of proinflammatory pathways and the activation of injury repair-related pathways may be critical for the protective effects of CLA in ruminal epithelium.

In addition to inflammation regulation, PPAR is also known for its role in regulating gene networks involved in lipid metabolism (Yuan et al., 2015). Our results revealed that the expression of genes involved in lipolysis including PLIN2 (Takahashi et al., 2016), ANGPTL4 (McQueen et al., 2017) and ABHD5 (Sanders et al., 2017), as well as the CPT1A, which is a rate-limiting enzyme in the fatty acid β-oxidation process (Raud et al., 2018), were significantly increased in ruminal epithelial cells pretreated with CLA, a finding consistent with those of previous studies showing that CLA plays a positive role in lipid lipolysis and fatty acid oxidation (Lehnen et al., 2015). Emerging evidence indicates that lipid metabolism and the immune response are coordinately regulated at multiple levels in the body (Di Cara et al., 2017). Kalucka et al. (2018) found that endothelial loss of fatty acid oxidation promoted leukocyte infiltration and barrier disruption of endothelial cells by increasing endothelial oxidative stress. Increased CPT1 expression was reported to correlate with relieved lipid-induced inflammatory responses (Jung et al., 2018). In addition, The positive role of ABHD5 as an intracellular lipolytic activator that was required for the generation of signaling lipids in response to inflammatory stimuli and even played a critical role in alleviating LPS-induced inflammatory responses has also been reported (Lord et al., 2012). Deficiency of ABHD5 was indicated to cause lipid overload in most cells and activate the macrophage NLRP3 inflammasome to promote IL-1β secretion, leading to a positive IL-1β-SOCS3-FOXO1-IL-1β feedback loop that enhances chronic inflammation (Miao et al., 2015). Furthermore, the protective effect of ANGPTL4 on proinflammatory processes has also been certified. Phua et al. (2017) found that colonic epithelial cell-secreted ANGPTL4 can act as a prospective regulator in altering the chemokine landscape in the colon to affect downstream inflammation. Our results also revealed an increased expression of SREBF1 in RECs after the CLA pretreatment. Although SREBF1 is involved in the process of fatty acid synthesis, it can drive the production of anti-inflammatory unsaturated fatty acids and was indicated to involve in the resolution of proinflammatory TLR4 signaling (Oishi et al., 2017). Shi et al. (2017) also found that the supplementation of CLA increased the production of unsaturated fatty acids and decreased the production of short- and medium-chain fatty acids in the milk of dairy goats, as well as increased the gene expression of SCD (stearoyl-CoA desaturase) that is involved in the biosynthesis of unsaturated fatty acids in the mammary tissues. These results in our study suggested a dual role of CLA in controlling lipid metabolism and immunoregulation, and a significantly enhanced adipocytokine signaling was also observed in our study, but the accurate regulation mechanism should be verified by measuring lipid metabolites and analyzing them together with the changes in cytokines in future studies.

**FIGURE 8:**
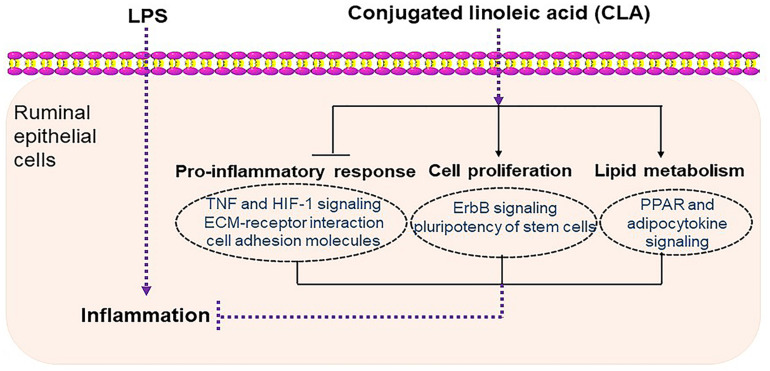
Schematic diagram summarizing the mechanisms underlying the protective effect of CLA against LPS-induced inflammation in RECs. CLA alleviated ruminal epithelial cell inflammation by inhibiting the proinflammatory process and by regulating cell proliferation and lipid metabolism, which are potentially related to injury repair and the immune response.

In conclusion, our study demonstrated that CLA alleviated ruminal epithelial cell inflammation by inhibiting the production of proinflammatory cytokines and by regulating cell proliferation and lipid metabolism, which are potentially related to injury repair and the immune response (Figure 8). These findings provide novel insights to better understand the mechanisms underlying the protective innate immunity of CLA in ruminal epithelium and offer more information for understanding the cross talk of ruminal microbiota with innate immune cells in the ruminal epithelium.

## Data Availability Statement

The datasets presented in this study can be found in online repositories. The names of the repository/repositories and accession number(s) can be found in the article/Supplementary Material.

## Ethics Statement

The animal study was reviewed and approved by Institutional Animal Care and Use Committee of Zhejiang University of Technology.

## Author Contributions

CY conceived and designed the experiments, wrote the manuscript. CY, WL, SY, and BZ performed all the experiments and analyzed the data. ZF suggested a modification. All authors contributed to the article and approved the submitted version.

## Conflict of Interest

The authors declare that the research was conducted in the absence of any commercial or financial relationships that could be construed as a potential conflict of interest.

## Footnotes

1 ftp://ftp.ensembl.org/pub/release-95/fasta/ovis_aries/ and ftp://ftp.ensembl.org/pub/release-95/gtf/ovis_aries/

2 http://string-db.org/

## Abbreviations

ABHD5: abhydrolase domain-containing protein 5
ANGPTL4: angiopoietin-like 4
AP1: activator protein 1
AREG: amphiregulin
CAMs: cell adhesion molecules
CLA: conjugated linoleic acid
CPT1A: carnitine *O*-palmitoyltransferase 1
CX3CL1: C-X 3-C motif chemokine 1
DEGs: differentially expressed genes
ECM: extracellular matrix
EDN1: endothelin-1
EREG: epiregulin
FC: fold change
FGF: fibroblast growth factor
FPKM: fragments per kilobase of transcript per million fragments mapped
GO: gene ontology
HBEGF: heparin-binding EGF-like growth factor
ICAM1: intercellular adhesion molecule 1
IRF1: interferon regulatory factor 1
KEGG: Kyoto Encyclopedia of Genes and Genomes
LPS: lipopolysaccharide
PLIN2: perilipin-2
PPAR: peroxisome proliferator activated receptor
PPI: protein-protein interaction
RECs: ruminal epithelial cells
SPRY2: protein sprouty homolog 2
SREBF1: sterol regulatory element-binding transcription factor 1.

## References

[B1] Aschenbach J. R. Zebeli Q. Patra A. K. Greco G. Amasheh S. Penner G. B. (2019). Symposium review: the importance of the ruminal epithelial barrier for a healthy and productive cow. *J. Dairy Sci.* 102 1866–1882. 10.3168/jds.2018-15243 30580938

[B2] Avula L. R. Knapen D. Buckinx R. Vergauwen L. Adriaensen D. Van Nassauw L. (2012). Whole-genome microarray analysis and functional characterization reveal distinct gene expression profiles and patterns in two mouse models of ileal inflammation. *BMC Genomics* 13:377. 10.1186/1471-2164-13-377 22866923 PMC3599598

[B3] Ayuk S. M. Abrahamse H. Houreld N. N. (2016). The role of photobiomodulation on gene expression of cell adhesion molecules in diabetic wounded fibroblasts in vitro. *J. Photochem. Photobiol. B Biol.* 161 368–374. 10.1016/j.jphotobiol.2016.05.027 27295416

[B4] Basiricò L. Morera P. Dipasquale D. Tröscher A. Bernabucci U. (2017). Comparison between conjugated linoleic acid and essential fatty acids in preventing oxidative stress in bovine mammary epithelial cells. *J. Dairy Sci.* 100 2299–2309. 10.3168/jds.2016-11729 28088424

[B5] Bradley J. R. (2008). TNF-mediated inflammatory disease. *J. Pathol.* 214 149–160. 10.1002/path.2287 18161752

[B6] Bruen R. Fitzsimons S. Belton O. (2017). Atheroprotective effects of conjugated linoleic acid. *Br. J. Clin. Pharmacol.* 83 46–53. 10.1111/bcp.12948 27037767 PMC5338159

[B7] Changhua L. Jindong Y. Defa L. Lidan Z. Shiyan Q. Jianjun X. (2005). Conjugated linoleic acid attenuates the production and gene expression of proinflammatory cytokines in weaned pigs challenged with lipopolysaccharide. *J. Nutr*. 135 239–244. 10.1093/jn/135.2.239 15671220

[B8] Chen Y. Yang B. Ross R. P. Jin Y. Stanton C. Zhao J. (2019). Orally administered CLA ameliorates DSS-induced colitis in mice via intestinal barrier improvement, oxidative stress reduction, and inflammatory cytokine and gut microbiota modulation. *J. Agric. Food Chem.* 67 13282–13298. 10.1021/acs.jafc.9b05744 31690068

[B9] Coelho O. G. L. Cândido F. G. Alfenas R. C. G. (2019). Dietary fat and gut microbiota: mechanisms involved in obesity control. *Crit. Rev. Food Sci. Nutr.* 59 3045–3053. 10.1080/10408398.2018.1481821 29851507

[B10] Daynes R. A. Jones D. C. (2002). Emerging roles of PPARs in inflammation and immunity. *Nat. Rev. Immunol.* 2 748–759. 10.1038/nri912 12360213

[B11] Denisenko E. Guler R. Mhlanga M. Suzuki H. Brombacher F. Schmeier S. (2019). Transcriptionally induced enhancers in the macrophage immune response to Mycobacterium tuberculosis infection. *BMC Genomics* 20:71. 10.1186/s12864-019-5450-6 30669987 PMC6341744

[B12] Di Cara F. Sheshachalam A. Braverman N. E. Rachubinski R. A. Simmonds A. J. (2017). Peroxisome-mediated metabolism is required for immune response to microbial infection. *Immunity* 47 93–106. 10.1016/j.immuni.2017.06.016 28723556

[B13] Dignazio L. Bandarra D. Rocha S. (2016). NF-κB and HIF crosstalk in immune responses. *FEBS J.* 283 413–424. 26513405 10.1111/febs.13578 PMC4864946

[B14] Ernandez T. Mayadas T. N. (2009). Immunoregulatory role of TNFalpha in inflammatory kidney diseases. *Kidney Int.* 76 262–276. 10.1038/ki.2009.142 19436333

[B15] Garibay-Nieto N. Queipo-García G. Alvarez F. Bustos M. Villanueva E. Ramírez F. (2017). Effects of conjugated linoleic acid and metformin on insulin sensitivity in obese children: randomized clinical trial. *J. Clin. Endocrinol. Metab.* 102 132–140. 10.1210/jc.2016-2701 27778642

[B16] Guo D. Xiao L. Hu H. Liu M. Yang L. Lin X. (2018). FGF21 protects human umbilical vein endothelial cells against high glucose-induced apoptosis via PI3K/Akt/Fox3a signaling pathway. *J. Diabetes Complicat.* 32 729–736. 10.1016/j.jdiacomp.2018.05.012 29907326

[B17] Halligan D. N. Murphy S. J. Taylor C. T. (2016). The hypoxia-inducible factor (HIF) couples immunity with metabolism. *Semin Immunol.* 28 469–477. 10.1016/j.smim.2016.09.004 27717536

[B18] Han J. Shao J. Chen Q. Sun H. Guan L. Li Y. (2019). Transcriptional changes in the hypothalamus, pituitary, and mammary gland underlying decreased lactation performance in mice under heat stress. *Faseb J.* 33 12588–12601. 10.1096/fj.201901045R 31480864 PMC6902726

[B19] Iizuka M. Konno S. (2011). Wound healing of intestinal epithelial cells. *World J Gastroenterol.* 17 2161–2171. 10.3748/wjg.v17.i17.2161 21633524 PMC3092866

[B20] Jones S. A. Jenkins B. J. (2018). Recent insights into targeting the IL-6 cytokine family in inflammatory diseases and cancer. *Nat. Rev. Immunol.* 18 773–789. 10.1038/s41577-018-0066-7 30254251

[B21] Jung T. W. Lee S. H. Kim H. C. Bang J. S. Abd El-Aty A. M. Hacımüftüoǧlu A. (2018). METRNL attenuates lipid-induced inflammation and insulin resistance via AMPK or PPARδ-dependent pathways in skeletal muscle of mice. *Exp. Mol. Med.* 50:122. 10.1038/s12276-018-0147-5 30213948 PMC6137187

[B22] Kalucka J. Bierhansl L. Conchinha N. V. Missiaen R. Elia I. Brüning U. (2018). Quiescent endothelial cells upregulate fatty acid β-oxidation for vasculoprotection via redox homeostasis. *Cell Metab.* 28 881–894. 10.1016/j.cmet.2018.07.016 30146488

[B23] Kamanga-Sollo E. Thornton K. J. White M. E. Dayton W. R. (2014). Role of G protein-coupled estrogen receptor-1, matrix metalloproteinases 2 and 9, and heparin binding epidermal growth factor-like growth factor in estradiol-17β-stimulated bovine satellite cell proliferation. *Domest. Anim. Endocrinol.* 49 20–26. 10.1016/j.domaniend.2014.04.004 25010024

[B24] Kataria H. Alizadeh A. Karimi-Abdolrezaee S. (2019). Neuregulin-1/ErbB network: an emerging modulator of nervous system injury and repair. *Prog Neurobiol.* 180:101643. 10.1016/j.pneurobio.2019.101643 31229498

[B25] Kim J. H. Kim Y. Kim Y. J. Park Y. (2016). Conjugated linoleic acid: potential health benefits as a functional food ingredient. *Annu. Rev. Food Sci. Technol.* 7 221–244. 10.1146/annurev-food-041715-033028 26735796

[B26] Kim J. T. Li C. Weiss H. L. Zhou Y. Liu C. Wang Q. (2019). Regulation of ketogenic enzyme HMGCS2 by Wnt/β-catenin/PPARγ pathway in intestinal cells. *Cells* 8:106. 10.3390/cells8091106 31546785 PMC6770209

[B27] Klotz J. L. Baldwin R. L. T. Gillis R. C. Heitmann R. N. (2001). Refinements in primary rumen epithelial cell incubation techniques. *J. Dairy Sci.* 84 183–193. 10.3168/jds.S0022-0302(01)74468-2 11210032

[B28] Ko J. H. Kim H. J. Jeong H. J. Lee H. J. Oh J. Y. (2020). Mesenchymal stem and stromal cells harness macrophage-derived amphiregulin to maintain tissue homeostasis. *Cell Rep.* 30 3806–3820. 10.1016/j.celrep.2020.02.062 32187551

[B29] Langlais D. Barreiro L. B. Gros P. (2016). The macrophage IRF8/IRF1 regulome is required for protection against infections and is associated with chronic inflammation. *J. Exp. Med.* 213 585–603. 10.1084/jem.20151764 27001747 PMC4821649

[B30] Lehnen T. E. da Silva M. R. Camacho A. Marcadenti A. Lehnen A. M. (2015). A review on effects of conjugated linoleic fatty acid (CLA) upon body composition and energetic metabolism. *J. Int. Soc. Sports Nutr.* 12:36. 10.1186/s12970-015-0097-4 26388708 PMC4574006

[B31] Liu M. Zhang Z. Sampson L. Zhou X. Nalapareddy K. Feng Y. (2017). RHOA GTPase controls YAP-mediated EREG signaling in small intestinal stem cell maintenance. *Stem Cell Rep.* 9 1961–1975. 10.1016/j.stemcr.2017.10.004 29129684 PMC5785633

[B32] Lord C. C. Betters J. L. Ivanova P. T. Milne S. B. Myers D. S. Madenspacher J. (2012). CGI-58/ABHD5-derived signaling lipids regulate systemic inflammation and insulin action. *Diabetes* 61 355–363. 10.2337/db11-0994 22228714 PMC3266405

[B33] Mao S. Y. Huo W. J. Zhu W. Y. (2016). Microbiome-metabolome analysis reveals unhealthy alterations in the composition and metabolism of ruminal microbiota with increasing dietary grain in a goat model. *Environ. Microbiol.* 18 525–541. 10.1111/1462-2920.12724 25471302

[B34] Masur F. Benesch F. Pfannkuche H. Fuhrmann H. Gäbel G. (2016). Conjugated linoleic acids influence fatty acid metabolism in ovine ruminal epithelial cells. *J. Dairy Sci.* 99 3081–3095. 10.3168/jds.2015-10042 26830749

[B35] McQueen A. E. Kanamaluru D. Yan K. Gray N. E. Wu L. Li M. L. (2017). The C-terminal fibrinogen-like domain of angiopoietin-like 4 stimulates adipose tissue lipolysis and promotes energy expenditure. *J. Biol. Chem*. 292 16122–16134. 10.1074/jbc.M117.803973 28842503 PMC5625043

[B36] Miao H. Ou J. Zhang X. Chen Y. Xue B. Shi H. (2015). Macrophage CGI-58 deficiency promotes IL-1β transcription by activating the SOCS3-FOXO1 pathway. *Clin. Sci. (Lond).* 128 493–506. 10.1042/cs20140414 25431838

[B37] Minuti A. Palladino A. Khan M. J. Alqarni S. Agrawal A. Piccioli-Capelli F. (2015). Abundance of ruminal bacteria, epithelial gene expression, and systemic biomarkers of metabolism and inflammation are altered during the peripartal period in dairy cows. *J. Dairy Sci.* 98 8940–8951. 10.3168/jds.2015-9722 26409956

[B38] Mohammadi I. Mahdavi A. H. Rabiee F. Nasr Esfahani M. H. Ghaedi K. (2020). Positive effects of conjugated linoleic acid (CLA) on the PGC1-α expression under the inflammatory conditions induced by TNF-α in the C2C12 cell line. *Gene* 735:144394. 10.1016/j.gene.2020.144394 31987906

[B39] Moreira T. G. Horta L. S. Gomes-Santos A. C. Oliveira R. P. Queiroz N. Mangani D. (2019). CLA-supplemented diet accelerates experimental colorectal cancer by inducing TGF-β-producing macrophages and T cells. *Mucosal. Immunol.* 12 188–199. 10.1038/s41385-018-0090-8 30279515

[B40] Murch S. H. Braegger C. P. Sessa W. C. MacDonald T. T. (1992). High endothelin-1 immunoreactivity in Crohn’s disease and ulcerative colitis. *Lancet* 339 381–385. 10.1016/0140-6736(92)90077-g 1346658

[B41] Oishi Y. Spann N. J. Link V. M. Muse E. D. Strid T. Edillor C. (2017). SREBP1 contributes to resolution of pro-inflammatory TLR4 signaling by reprogramming fatty acid metabolism. *Cell Metab.* 25 412–427. 10.1016/j.cmet.2016.11.009 28041958 PMC5568699

[B42] Oktar B. K. Coşkun T. Bozkurt A. Yegen B. C. Yüksel M. Haklar G. (2000). Endothelin-1-induced PMN infiltration and mucosal dysfunction in the rat small intestine. *Am. J. Physiol. Gastrointest. Liver Physiol.* 279 G483–G491. 10.1152/ajpgi.2000.279.3.G483 10960346

[B43] Pellattiero E. Cecchinato A. Tagliapietra F. Schiavon S. Bittante G. (2015). The use of 2-dimensional gas chromatography to investigate the effect of rumen-protected conjugated linoleic acid, breed, and lactation stage on the fatty acid profile of sheep milk. *J. Dairy Sci.* 98 2088–2102. 10.3168/jds.2014-8395 25648807

[B44] Peyssonnaux C. Cejudo-Martin P. Doedens A. Zinkernagel A. S. Johnson R. S. Nizet V. (2007). Cutting edge: Essential role of hypoxia inducible factor-1alpha in development of lipopolysaccharide-induced sepsis. *J. Immunol.* 178 7516–7519. 10.4049/jimmunol.178.12.7516 17548584

[B45] Phua T. Sng M. K. Tan E. H. Chee D. S. Li Y. Wee J. W. (2017). Angiopoietin-like 4 mediates colonic inflammation by regulating chemokine transcript stability via tristetraprolin. *Sci. Rep.* 7:44351. 10.1038/srep44351 28287161 PMC5347094

[B46] Pinto D. Marzani B. Minervini F. Calasso M. Giuliani G. Gobbetti M. (2011). Plantaricin A synthesized by *Lactobacillus plantarum* induces *in vitro* proliferation and migration of human keratinocytes and increases the expression of TGF-β1, FGF7, VEGF-A and IL-8 genes. *Peptides* 32 1815–1824. 10.1016/j.peptides.2011.07.004 21782870

[B47] Qin N. Bayat A. R. Trevisi E. Minuti A. Kairenius P. Viitala S. (2018). Dietary supplement of conjugated linoleic acids or polyunsaturated fatty acids suppressed the mobilization of body fat reserves in dairy cows at early lactation through different pathways. *J. Dairy Sci.* 101 7954–7970. 10.3168/jds.2017-14298 29960784

[B48] Rai M. F. Tycksen E. D. Sandell L. J. Brophy R. H. (2018). Advantages of RNA-seq compared to RNA microarrays for transcriptome profiling of anterior cruciate ligament tears. *J. Orthop. Res.* 36 484–497. 10.1002/jor.23661 28749036 PMC5787041

[B49] Raud B. Roy D. G. Divakaruni A. S. Tarasenko T. N. Franke R. Ma E. H. (2018). Etomoxir actions on regulatory and memory T cells are independent of Cpt1a-mediated fatty acid oxidation. *Cell Metab*. 28 504–515. 10.1016/j.cmet.2018.06.002 30043753 PMC6747686

[B50] Saba F. Sirigu A. Pillai R. Caria P. Cordeddu L. Carta G. (2019). Downregulation of inflammatory markers by conjugated linoleic acid isomers in human cultured astrocytes. *Nutr. Neurosci*. 22 207–214. 10.1080/1028415X.2017.1367130 28847225

[B51] Sanders M. A. Zhang H. Mladenovic L. Tseng Y. Y. Granneman J. G. (2017). Molecular basis of ABHD5 lipolysis activation. *Sci. Rep*. 7:42589. 10.1038/srep42589 28211464 PMC5314347

[B52] Schiavon S. De Marchi M. Tagliapietra F. Bailoni L. Cecchinato A. Bittante G. (2011). Effect of high or low protein ration combined or not with rumen protected conjugated linoleic acid (CLA) on meat CLA content and quality traits of double-muscled Piemontese bulls. *Meat Sci.* 89 133–142. 10.1016/j.meatsci.2011.03.025 21561723

[B53] Shen H. Xu Z. Shen Z. Lu Z. (2019). The regulation of ruminal short-chain fatty acids on the functions of rumen barriers. *Front. Physiol.* 10:1305. 10.3389/fphys.2019.01305 31749707 PMC6842973

[B54] Shi H. Zhang T. Li C. Wang J. Huang J. Li Z. (2017). trans-10,cis-12-conjugated linoleic acid affects expression of lipogenic genes in mammary glands of lactating dairy goats. *J. Agric. Food Chem*. 65 9460–9467. 10.1021/acs.jafc.7b02377 29019657

[B55] Stammberger P. Baczako K. (1999). Cytokeratin 19 expression in human gastrointestinal mucosa during human prenatal development and in gastrointestinal tumours: relation to cell proliferation. *Cell Tissue Res.* 298 377–381. 10.1007/s004419900085 10571127

[B56] Takahashi Y. Shinoda A. Kamada H. Shimizu M. Inoue J. Sato R. (2016). Perilipin2 plays a positive role in adipocytes during lipolysis by escaping proteasomal degradation. *Sci Rep*. 6:20975. 10.1038/srep20975 26876687 PMC4753471

[B57] Venkatesh D. Ernandez T. Rosetti F. Batal I. Cullere X. Luscinskas F. W. (2013). Endothelial TNF receptor 2 induces IRF1 transcription factor-dependent interferon-β autocrine signaling to promote monocyte recruitment. *Immunity* 38 1025–1037. 10.1016/j.immuni.2013.01.012 23623383 PMC3760474

[B58] Viladomiu M. Hontecillas R. Bassaganya-Riera J. (2016). Modulation of inflammation and immunity by dietary conjugated linoleic acid. *Eur. J. Pharmacol.* 785 87–95. 10.1016/j.ejphar.2015.03.095 25987426

[B59] Ward-Kavanagh L. K. Lin W. W. Šedý J. R. Ware C. F. (2016). The TNF receptor superfamily in co-stimulating and co-inhibitory responses. *Immunity* 44 1005–1019. 10.1016/j.immuni.2016.04.019 27192566 PMC4882112

[B60] Wight T. N. Frevert C. W. Debley J. S. Reeves S. R. Parks W. C. Ziegler S. F. (2017). Interplay of extracellular matrix and leukocytes in lung inflammation. *Cell. Immunol.* 312 1–14. 10.1016/j.cellimm.2016.12.003 28077237 PMC5290208

[B61] Yan X. Managlia E. Tan X. D. De Plaen I. G. (2019). Prenatal inflammation impairs intestinal microvascular development through a TNF-dependent mechanism and predisposes newborn mice to necrotizing enterocolitis. *Am. J. Physiol. Gastrointest. Liver Physiol.* 317 G57–G66. 10.1152/ajpgi.00332.2018 31125264 PMC6689733

[B62] Yoshioka M. Takenouchi T. Kitani H. Okada H. Yamanaka N. (2016). Establishment of SV40 large T antigen-immortalized bovine liver sinusoidal cell lines and their immunological responses to deoxynivalenol and lipopolysaccharide. *Cell Biol. Int.* 40 1372–1379. 10.1002/cbin.10682 27624824

[B63] Yuan G. Chen X. Li D. (2015). Modulation of peroxisome proliferator-activated receptor gamma (PPAR γ) by conjugated fatty acid in obesity and inflammatory bowel disease. *J. Agric. Food Chem.* 63 1883–1895. 10.1021/jf505050c 25634802

[B64] Zhan K. Lin M. Liu M.-M. Sui Y.-N. Zhao G.-Q. (2017). Establishment of primary bovine intestinal epithelial cell culture and clone method. *In Vitro Cell. Dev. Biol. Anim.* 53 54–57. 10.1007/s11626-016-0082-5 27561890

[B65] Zhao Q. Chen S. Zhu Z. Yu L. Ren Y. Jiang M. (2018). miR-21 promotes EGF-induced pancreatic cancer cell proliferation by targeting Spry2. *Cell Death Dis.* 9:1157. 10.1038/s41419-018-1182-9 30464258 PMC6249286

