# Supplementary material for: Transcriptomic Analyses Reveal the Protective Immune Regulation of Conjugated Linoleic Acids in Sheep Ruminal Epithelial Cells
Source: Front Physiol. 2020 Oct 29;11:588082. doi: 10.3389/fphys.2020.588082 (PMC7658390; doi:10.3389/fphys.2020.588082)
Supplement: Supplementary file 1 [file Data_Sheet_1.docx]

**Transcriptomic Analyses Reveal the Protective Immune Regulation of Conjugated Linoleic Acids in Sheep Ruminal Epithelial Cells**

**Yang et al. Online Supplementary Material**

**Supplementary Table 1.** The structure of SV40.

| **Vector name** | SV40 overexpression lentivirus | |
| --- | --- | --- |
| **Vector information** | **Carrier element information** | EF1α-SV40-IRES-puromycin |
|  | **Resistance gene marker** | Puromycin |
| **Vector map** | 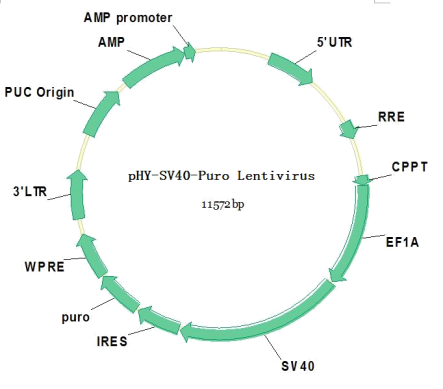 | |
| **The target sequence information of the carrier is as follows:**  gtggttcaaagtttttttcttccatttcaggtgtcgtgaggatctatttccggtgaattcatggataaagttttaaacagagaggaatctttgcagctaatggaccttctaggtcttgaaaggagtgcctgggggaatattcctctgatgagaaaggcatatttaaaaaaatgcaaggagtttcatcctgataaaggaggagatgaagaaaaaatgaagaaaatgaatactctgtacaagaaaatggaagatggagtaaaatatgctcatcaacctgactttggaggcttctgggatgcaactgagattccaacctatggaactgatgaatgggagcagtggtggaatgcctttaatgaggaaaacctgttttgctcagaagaaatgccatctagtgatgatgaggctactgctgactctcaacattctactcctccaaaaaagaagagaaaggtagaagaccccaaggactttccttcagaattgctaagttttttgagtcatgctgtgtttagtaatagaactcttgcttgctttgctatttacaccacaaaggaaaaagctgcactgctatacaagaaaattatggaaaaatattctgtaacctttataagtaggcataacagttataatcataacatactgttttttcttactccacacaggcatagagtgtctgctattaataactatgctcaaaaattgtgtacctttagctttttaatttgtaaaggggttaataaggaatatttgatgtatagtgccttgactagagatccattttctgttattgaggaaagtttgccaggtgggttaaaggagcatgattttaatccagaagaagcagaggaaactaaacaagtgtcctggaagcttgtaacagagtatgcaatggaaacaaaatgtgatgatgtgttgttattgcttgggatgtacttggaatttcagtacagttttgaaatgtgtttaaaatgtattaaaaaagaacagcccagccactataagtaccatgaaaagcattatgcaaatgctgctatatttgctgacagcaaaaaccaaaaaaccatatgccaacaggctgttgatactgttttagctaaaaagcgggttgatagcctacaattaactagagaacaaatgttaacaaacagatttaatgatcttttggataggatggatataatgtttggttctacaggctctgctgacatagaagaatggatggctggagttgcttggctacactgtttgttgcccaaaatggattcagtggtgtatgactttttaaaatgcatggtgtacaacattcctaaaaaaagatactggctgtttaaaggaccaattgatagtggtaaaactacattagcagctgctttgcttgaattatgtggggggaaagctttaaatgttaatttgcccttggacaggctgaactttgagctaggagtagctattgaccagtttttagtagtttttgaggatgtaaagggcactggaggggagtccagagatttgccttcaggtcagggaattaataacctggacaatttaagggattatttggatggcagtgttaaggtaaacttagaaaagaaacacctaaataaaagaactcaaatatttccccctggaatagtcaccatgaatgagtacagtgtgcctaaaacactgcaggccagatttgtaaaacaaatagattttaggcccaaagattatttaaagcattgcctggaacgcagtgagtttttgttagaaaagagaataattcaaagtggcattgctttgcttcttatgttaatttggtacagacctgtggctgagtttgctcaaagtattcagagcagaattgtggagtggaaagagagattggacaaagagtttagtttgtcagtgtatcaaaaaatgaagtttaatgtggctatgggaattggagttttagattggctaagaaacagtgatgatgatgatgaagacagccaggaaaatgctgataaaaatgaagatggtggggagaagaacatggaagactcagggcatgaaacaggcattgattcacagtcccaaggctcatttcaggcccctcagtcctcacagtctgttcatgatcataatcagccataccacatttgtagaggttttacttgctttaaaaaacctcccacacctccccctgaacctgaaacagagcaaaagctcatttctgaagaggacttgtaatctagacacagtgcagcactctcaacgttcaaggacactacgcgtctggaacaatcaacc  **The yellow region is the target sequence region** | | |

**Supplementary Table 2.** Primers used in mRNA abundance analysis.

| Gene | NCBI accession | Primer sequence 5’- 3’ |
| --- | --- | --- |
| TNF-α | NM_001024860.1 | F: GTCCAACTTTAAACAGCTGCACTTA  R: CCCAAACTTGTGGACCCGA |
| IL-6 | NM_001009392.1 | F: ACACTGACATGCTGGAGAAGATGC  R: CCGAATAGCTCTCAGGCTGAACTG |
| IL-8 | KC912524.1 | F: ATGACTTCCAAGCTGGCTGTTG  R: TTGATAAATTTGGGGTGGAAAG |
| NF-κB | XM_012119625 | F: ATTGAGCGTCCTGTAACCGTGTTC  R: TGCACCTCCTCCTTGTCTTCTACC |
| YWHAZ | NM_001267887.1 | F: GATGAAGCCATTGCTGAACTTGA  R: CAGCTTCGTCTCCTTGGGTA |
| GADPH | NM_001190390.1 | F: GTCTTCACTACCATGGAGAAGG  R: TCATGGATGACCTTGGCCAG |


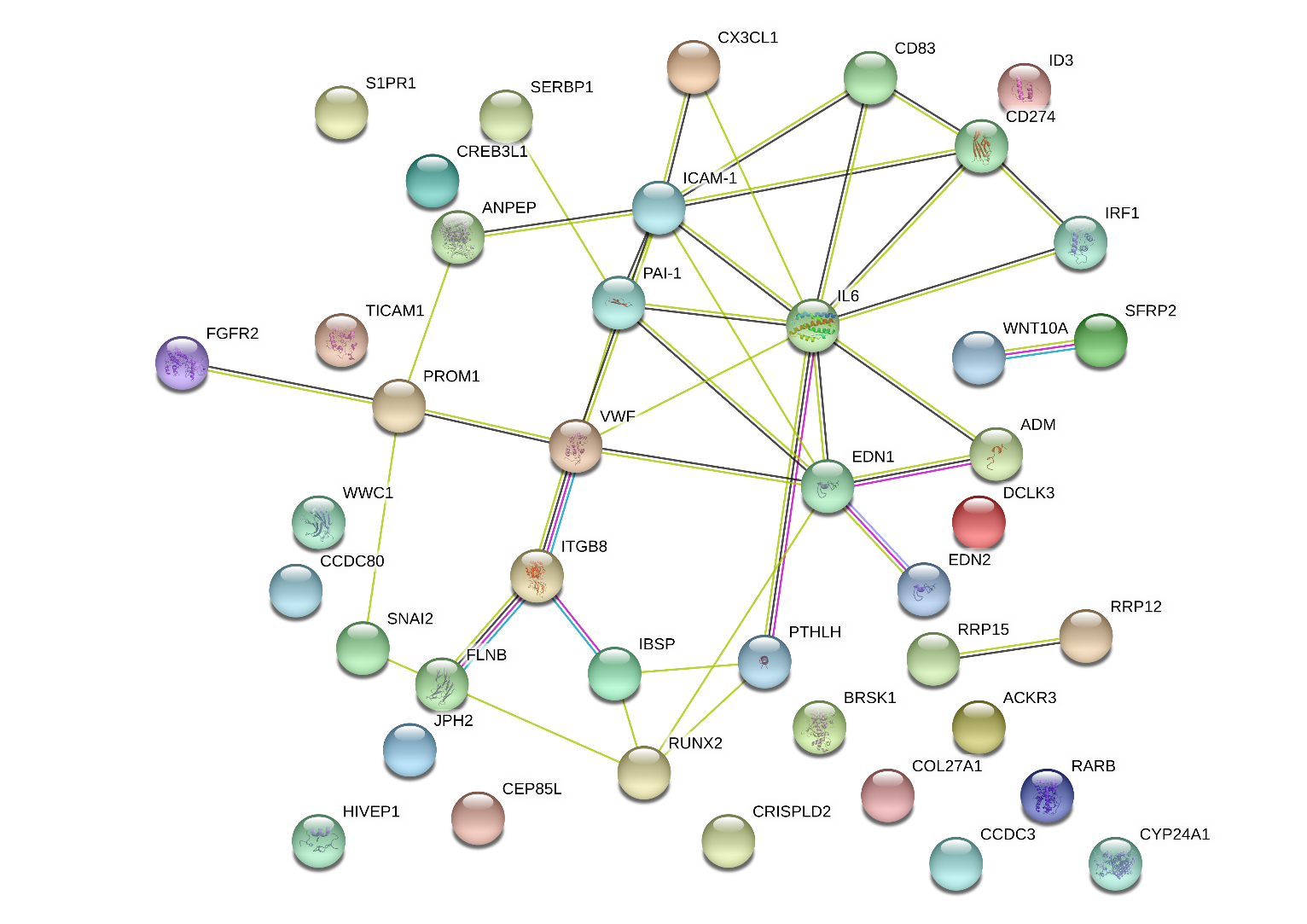


**Supplementary Figure 1.** PPI network of the overlapping DEGs that were downregulated in the CLA+LPS group and upregulated in the LPS group. All the nodes and edges were presented.


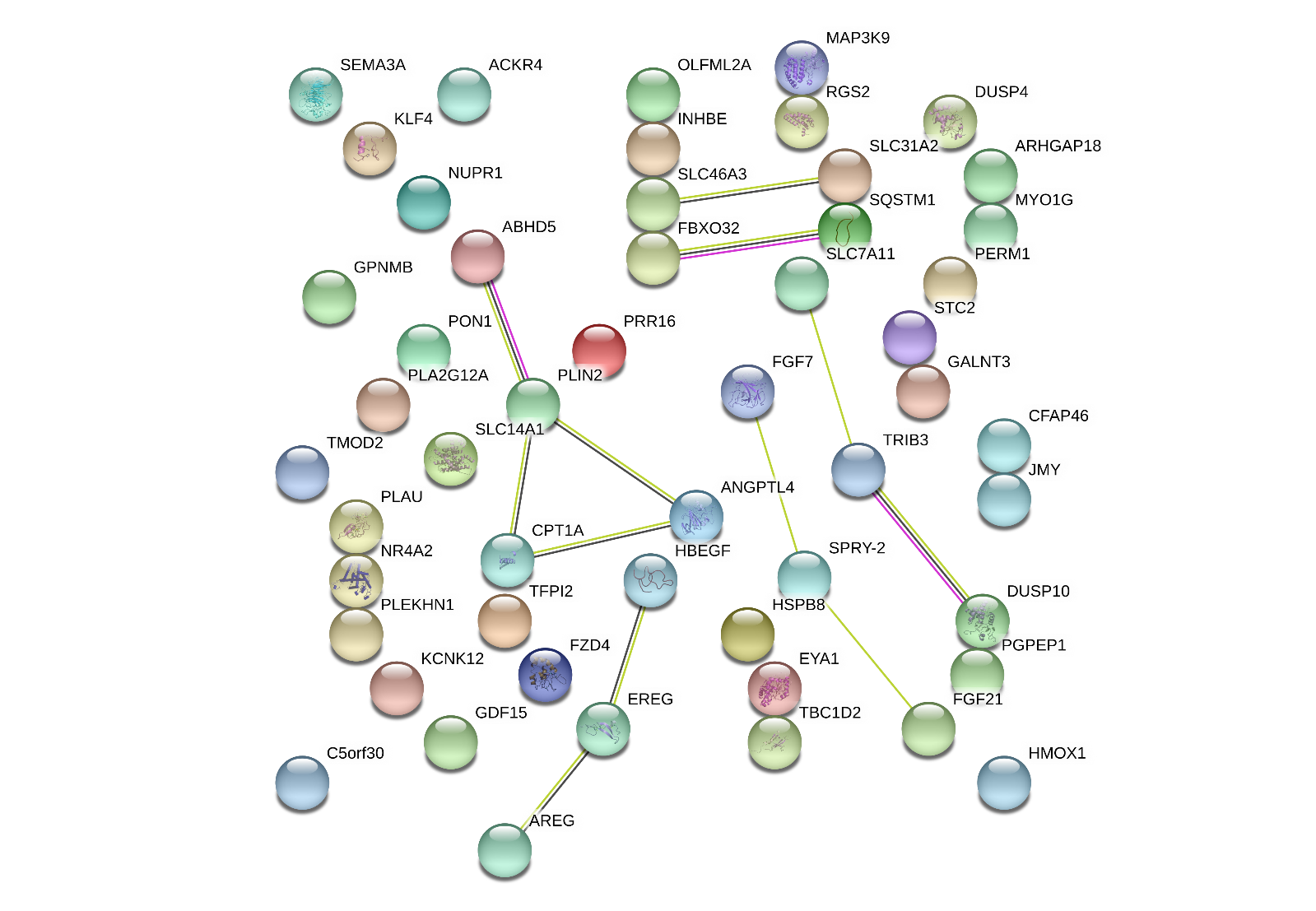


**Supplementary Figure 2.** PPI network of the DEGs that were upregulated in the CLA+LPS group compared with the expression level in the LPS group. All the nodes and edges were presented.
